# Endoscopic and surgical management of Bouveret’s syndrome complicated by gallstone ileus

**DOI:** 10.1093/jscr/rjab464

**Published:** 2021-10-26

**Authors:** Jaya Sai Varre, Jin Ling Wu, Peter Hopmann, Oscar Ruiz, Raghuram Reddy

**Affiliations:** Department of General Surgery, OhioHealth Riverside Methodist Hospital, 3535 Olentangy River Road, Columbus, OH 43214, USA; Department of General Surgery, OhioHealth Riverside Methodist Hospital, 3535 Olentangy River Road, Columbus, OH 43214, USA; Department of General Surgery, OhioHealth Riverside Methodist Hospital, 3535 Olentangy River Road, Columbus, OH 43214, USA; Department of General Surgery, OhioHealth Riverside Methodist Hospital, 3535 Olentangy River Road, Columbus, OH 43214, USA; Department of Medical Education, OhioHealth Riverside Methodist Hospital, 3535 Olentangy River Road, Columbus, OH 43214, USA; Ohio Gastroenterology Group, Inc., OhioHealth Riverside Methodist Hospital, 3400 Olentangy River Road, Columbus, OH 43202, USA

## Abstract

Bouveret’s syndrome is a rare complication that occurs most commonly in elderly patients with multiple comorbidities. It is secondary to an impacted gallstone causing gastric outlet obstruction from a cholecystoduodenal fistula, and there is no defined standardized management in current literature. A 92-year-old woman presents to our tertiary community hospital with abdominal discomfort concerning for bowel obstruction. Computed tomography revealed pneumobilia with a cholecystoduodenal fistula and a large gallstone in the proximal duodenum causing gastric outlet obstruction. The impacted gallstone failed endoscopic extraction with electrohydraulic lithotripsy, and patient subsequently developed distal gallstone ileus requiring exploratory laparotomy and enterolithotomy. This case report examines the need for early coordinated endoscopic and surgical management of a patient with Bouveret’s syndrome complicated by gallstone ileus as it is associated with high morbidity and mortality rates.

## INTRODUCTION

Bouveret’s syndrome is caused by formation of a cholecystoduodenal fistula from protracted biliary disease course where migrating gallstones can become impacted causing gastric outlet obstruction. Gallstone ileus occurs in 0.3–0.5% of patients presenting with cholelithiasis, and Bouveret’s syndrome represents only 1–3% of these cases [1, 2]. However, Bouveret’s syndrome has considerable morbidity and mortality rates ranging from 12 to 30% due to significant associated patient comorbidities [3, 4]. Due to the disease rarity, there have been no standardized recommendations for management with a wide variety of management techniques including endoscopic and surgical options. We review the importance of coordinated endoscopic and surgical management of a Bouveret’s syndrome complicated by gallstone ileus and its postoperative outcome at our institution.

## CASE REPORT

A 92-year-old woman presented to our tertiary hospital with 2 weeks of vague abdominal discomfort associated with nausea and vomiting. Past medical history includes hypertension, diabetes mellitus, heart failure, atrial fibrillation on coumadin and peripheral vascular disease. Patient reported she had an episode of acute cholecystitis managed with percutaneous cholecystostomy ~1 year ago that has since been removed.

**Figure 1:**
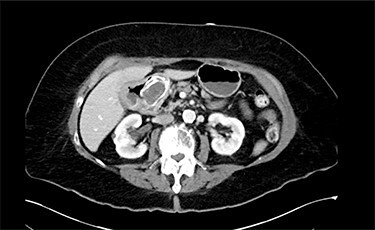
Abdominal CT scan (axial plane) showing gallstone in the duodenum with a cholecystoduodenal fistula.

**Figure 2:**
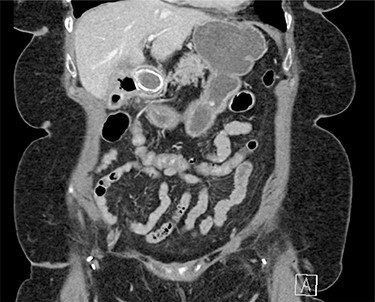
Abdominal CT scan (coronal plane) with the large impacted gallstone causing gastric outlet obstruction proximally with no small bowel dilation distally.

On physical exam, patient was tender in the epigastrium and right upper quadrant with no abdominal distension or peritoneal signs. Initial laboratory studies revealed white blood cell count: 8.83 K/mcl, international normalized ratio: 2.2, aspartate aminotransferase: 22 U/l, alanine aminotransferase: 19 U/l, total bilirubin 0.5 mg/dl and direct bilirubin: 0.1 mg/dl. Computed tomography (CT) scan of abdomen and pelvis showed pneumobilia with a cholecystoduodenal fistula and a large gallstone causing gastric distension proximally with no small bowel dilatation distally (Figs 1 and 2).

**Figure 3:**
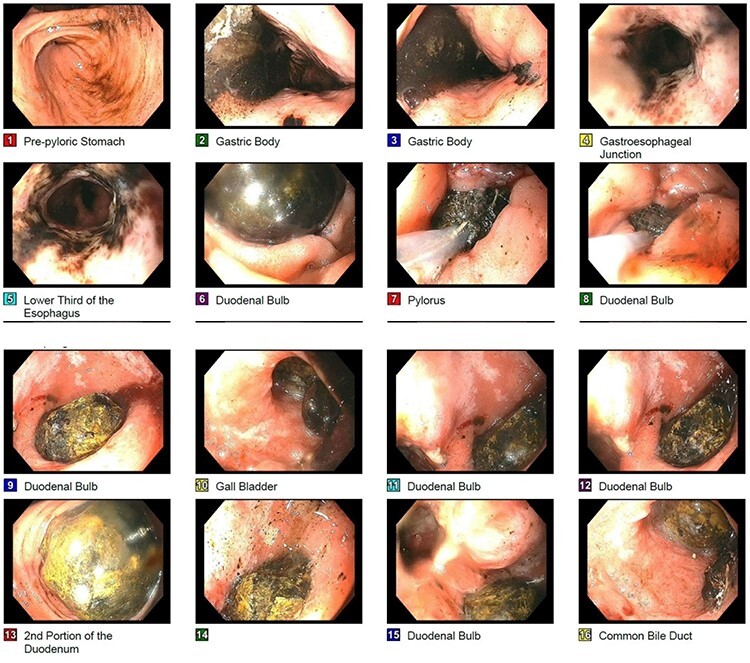
Endoscopic picture of large impacted gallstone in the duodenum with noted cholecystoduodenal fistula noted on image 15.

Patient was taken for esophagogastroduodenoscopy on hospital Day 2 and was found to have food residue in the gastric fundus and a large gallstone with fistula in the duodenal bulb (Fig. 3). Attempted retrieval with endoscopic basket and intracorporeal electrohydraulic lithotripsy (EHL) was unsuccessful due to inability to extract the stone past the pylorus and density of the stone, respectively. Subsequently, the patient had no bowel function and worsening abdominal distension concerning for developing gallstone ileus secondary to migration of the gallstone. After prolonged discussion with patient and family, the decision was to initiate total parenteral nutrition and proceed with an exploratory laparotomy on hospital Day 4.

**Figure 4:**
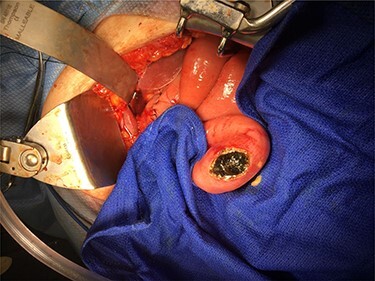
Distal gallstone causing gallstone ileus with proximal enterotomy for extraction.

**Figure 5:**
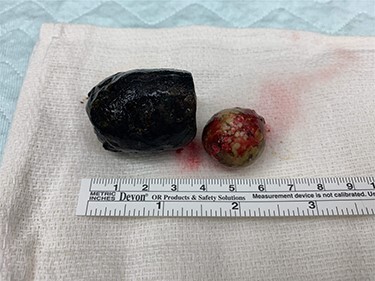
Larger gallstone on the left measuring 3.4 cm removed from distal small bowel. Smaller gallstone on the right measuring 2 cm removed from second portion of duodenum.

Laparotomy revealed significant cholecystoduodenal inflammation, a 2-cm gallstone impacting in the second portion of the duodenum (D2) and the 3.4 cm gallstone seen on endoscopy in the mid-portion of jejunum causing an obstruction. The gallstones were extracted through two separate enterotomies in the proximal duodenum and jejunum due to the degree of inflammation near the cholecystoduodenal fistula and the size of the gallstones (Figs 4 and 5). All enterotomy sites were closed in two layers with omental patch at the proximal duodenotomy site. A 10-French Jackson-Pratt drain was placed near the duodenotomy.

Postoperatively, her course was complicated by poor pain control, delirium and delayed return of bowel function. An upper gastrointestinal study was obtained on postoperative Day 6 that was negative for leak. On postoperative Day 7 the patient had worsening respiratory status and leukocytosis with repeat CT scan showing bilateral pleural effusions concerning for aspiration pneumonia. She was transferred to the surgical intensive care unit for respiratory management. At this point the family decided to transition to comfort care. She was discharged to hospice and passed on postoperative Day 12.

## DISCUSSION

The pathophysiology of Bouveret’s syndrome is related to chronic inflammation, impaired arterial flow or reduced venous drainage between the gallbladder and intestinal wall leading to significant adhesive disease and fistula formation [5]. Due to the significant morbidity and mortality of these cases, several authors have proposed initial management with endoscopy.

Initial endoscopic management is preferred due to decreased complications compared with open abdominal surgery and not requiring formal repair of the cholecystoduodenal fistula. The first successfully reported stone extraction was done endoscopically with a basket retrieval device, which has the greatest efficacy with smaller gallstones [2]. Larger stones have been treated at tertiary and quaternary care centers with EHL with or without laser therapy followed by subsequent retrieval of small stone fragments. EHL is performed by continuously irrigating and submerging the stone while delivering short pulses of high-voltage, which allows for immediate expansion of the surrounding liquid and creates a shock wave. This fragments the stone, allowing for partial removal into the gastric body where they can be further reduced via mechanical lithotripsy and then extracted [6]. Laser lithotripsy requires a small laser fiber penetrating and fragmenting the stone. This has been reported to be successful in several case reports; however, some patients required multiple endoscopic sessions. Important limitations of these therapies are increased risk for bowel injury, potential perforation and stone migration distally leading to gallstone ileus [7]. As a result, endoscopic approaches may be preferred in patients who are poor surgical candidates for an open enterolithotomy. However, with successful retrieval rates as low as 10%, endoscopy is closely followed by surgical extraction through a gastrotomy or duodenotomy [8].

Multiple studies describe two main approaches: stone extraction via enterolithotomy, gastrotomy or cholecystotomy followed by subsequent fistula repair and cholecystectomy compared with a one-stage approach where these procedures are performed in the same operation [4]. In one report where a patient presented with an impacted gallstone in the third portion of the duodenum, a surgeon performed a gastrojejunostomy and allowed the authors to bypass the duodenum without repairing the fistula [9]. Several authors have suggested performing stone extraction alone in poor surgical candidates. A cholecystoduodenal fistula may undergo spontaneous closure in patients with no residual stones and a patent cystic duct. This method is associated with lower mortality of 12% compared with 20–30% in combined cases where a fistula repair and cholecystectomy is concurrently performed [5]. On the contrary, some authors favor a one-stage approach in younger patients as these fistulas may develop further complications such as recurrence, persistent symptoms and increased risk for gallbladder malignancy.

## CONCLUSION

The high morbidity and mortality of Bouveret’s syndrome requires multidisciplinary coordination of care with initial management by advanced endoscopy followed by surgical intervention.

## CONSENT

Written informed consent was obtained from the patient or his next of kin/power of attorney for publication of the cases and accompanying images. A copy of the written consent is available for review by the editor of this journal.

## CONFLICT OF INTEREST STATEMENT

None declared.
